# Estimation of the methylation pattern distribution from deep sequencing data

**DOI:** 10.1186/s12859-015-0600-6

**Published:** 2015-05-06

**Authors:** Peijie Lin, Sylvain Forêt, Susan R Wilson, Conrad J Burden

**Affiliations:** 1 Mathematical Sciences Institute, Australian National University, Canberra, ACT 2601 Australia; 2 Research School of Biology, Australian National University, Canberra, ACT 2601 Australia; 3 School of Mathematics and Statistics, University of New South Wales, 2052 NSW Sydney, Australia

**Keywords:** DNA methylation, Bisulfite sequencing, DNA methylation patterns, Epiallele

## Abstract

**Background:**

Bisulphite sequencing enables the detection of cytosine methylation. The sequence of the methylation states of cytosines on any given read forms a methylation pattern that carries substantially more information than merely studying the average methylation level at individual positions. In order to understand better the complexity of DNA methylation landscapes in biological samples, it is important to study the diversity of these methylation patterns. However, the accurate quantification of methylation patterns is subject to sequencing errors and spurious signals due to incomplete bisulphite conversion of cytosines.

**Results:**

A statistical model is developed which accounts for the distribution of DNA methylation patterns at any given locus. The model incorporates the effects of sequencing errors and spurious reads, and enables estimation of the true underlying distribution of methylation patterns.

**Conclusions:**

Calculation of the estimated distribution over methylation patterns is implemented in the R Bioconductor package MPFE. Source code and documentation of the package are also available for download at http://bioconductor.org/packages/3.0/bioc/html/MPFE.html.

**Electronic supplementary material:**

The online version of this article (doi:10.1186/s12859-015-0600-6) contains supplementary material, which is available to authorized users.

## Background

Epigenetic regulations are involved in a broad range of biological processes, including development, tissue homeostasis, learning and memory, as well as various diseases such as obesity and cancer [1-3].

DNA methylation is one of the best studied epigenetic molecular mechanisms. It consists of the addition of a methyl group to the cytosine residues (C) of a DNA molecule. In animals, DNA methylation usually takes place in the CpG context: cytosines followed by a guanine (G) residue.

DNA methylation modulates gene expression through a variety of mechanisms. In vertebrates, methylation in the promoter region usually has a repressive effect on transcription initiation. By contrast, methylation of gene bodies is generally associated with an active transcriptional state and has been shown to play an important role in the control of alternative splicing [4,5].

The diverse and subtle effects of DNA methylation enable a given genome to produce different phenotypic outputs as part of a developmental program or in response to environmental factors. This has fundamental implications at the organismal level, where DNA methylation plays an important role in phenotypic plasticity [6]. This is also important at the cellular level to create diverse cell types, tissues and organs all based on the same genome. DNA methylation patterns can thus change from one cell type to another or within a cell under different conditions [7].

The diversity of methylation patterns in a sample can be studied with a single base pair resolution using the bisulphite sequencing technique [8]. When DNA is treated with bisulphite, the unmethylated cytosines are converted to uracils with a high (albeit not complete) efficiency, whereas the methylated cytosines remain as cytosines. A library is prepared from the bisulphite treated DNA by fragmenting to lengths of approximately 200 bp and PCR amplified. During this amplification process, uracils are replicated as thymines (T). The DNA library is then sequenced and the resulting reads are mapped to a reference. Within each read, CpG dinucleotides which have been converted to TpG are recognised as unmethylated, and unconverted CpG dinucleotides are recognised as methylated.

A common type of analysis carried out with this type of data is to estimate, for each cytosine, the global methylation level of a sample, namely, the average methylation level across all DNA strands in all the cells represented in that sample. This is usually done by correcting for the fact that bisulphite conversion is not a complete reaction (for instance, see [4,9]).

However, this type of analysis only considers the methylation level at individual positions and is oblivious to the fact that cytosines present on a given read represent a broader snapshot of the methylation landscape on a particular strand of DNA. One can therefore gain a significantly deeper insight into the complexity of DNA methylation landscapes by reconstituting the methylation patterns that are physically present on the same sequence, and thus come from the same cell. Each read can be seen as containing a small number of binary labelled CpG sites: 1 for methylated, 0 for unmethylated and represents the methylation pattern of a given strand of DNA in one particular cell. This approach is of particular interest when studying complex biological samples that contain a mixture of cell types, for instance a tumour or a whole insect brain. It gives a much more detailed picture about the diversity of DNA methylation in a sample than simply looking at the methylation level at each position.

The study of the diversity and dynamics of DNA methylation patterns has recently generated a great deal of interest as it allows assessment of the methylation status in subsets of cells within a sample. Such studies have provided novel insights into the role of DNA methylation in cell differentiation and reprogramming, and the evolution of tumours (for instance [10-12]). It has also been proposed that the DNA methylation patterns present in heterogeneous samples could be effective tumour-specific biomarkers [13].

The approach of looking at methylation patterns instead of individual CpG sites can be hampered by the lack of sequencing coverage depth. For reasons of cost, most whole genome bisulphite sequencing studies have a mean genomic coverage in the 10-100 range. If the underlying sample contains hundreds of methylation patterns, they cannot be sampled representatively. Consequently, studies looking at DNA methylation diversity have focussed on short patterns (around 4 CpGs) [12].

As an alternative, in order to reach a high coverage, researchers have often focussed on specific loci, either by sequencing PCR amplicons of bisulphite converted DNA, or by reduced representation bisulphite sequencing, or by using a capture assay (for review see [14]). In this paper, we focus on the analysis of amplicon data, but the statistical framework introduced here could be applied to data extracted from other types of sequencing approaches such as reduced representation and capture-based sequencing.

The problem of determining methylation patterns in the original sample from the observed data is non trivial. The purpose of this paper is to infer formally the probability distribution over all possible methylation profiles defined by the population of methylomes at a given locus. We show that taking into account the effect of the non-systematic errors of incomplete bisulphite conversion, as well as sequencing error, is critical as this removes a large number of spurious patterns present in the raw reads.

## Results and discussion

### Synthetic data

The probability distribution over methylation patterns at a given locus from from bisulphite sequencing data is estimated via an algorithm described below in the Methods section. The algorithm is implemented as an R Bioconductor [15] package MPFE (for **M**ethylation **P**atterns **F**requency **E**stimation).

As the true distribution over methylation patterns is always unknown in the laboratory, we have constructed a number of synthetic data sets to test the effectiveness of the algorithm. For a locus with *n* CpG sites we prescribe a true distribution *θ*_*k*_, *k*=1,…,2^*n*^, over the 2^*n*^ possible methylation patterns. We simulate from these patterns a set of *N*_read_ initial reads, each labelled with its ‘true’ methylation pattern, and then redistribute the reads among patterns according to the statistical model described in the Methods section to simulate errors due to incomplete bisulphite conversion and sequencing errors.

The rates of conversion and sequencing error can be estimated by spiking-in non-methylated DNA of known sequence, or by looking at positions known in advance not to be methylated [16].

Incomplete conversion is specified by a non-conversion rate parameter *ε* equal to the probability that an unmethylated cytosine will fail to convert. Its value is typically of order 10^−2^, and will be assumed in our simulations to be estimated independently from the rate of conversion of non-CpG cytosines. For instance, it could be calculated based on sequences known a priori not to be methylated. The sequencing error rates are specified by a vector *η*=(*η*_1_,…,*η*_*n*_), where *η*_*s*_∼*O*(10^−2^) is the probability that site *s* is methylated but registers as unmethylated and vice versa. These two sources of error can generate spurious methylation patterns, that is, patterns for which the observed read count *y*_*k*_ is non-zero when the true frequency of the pattern, *θ*_*k*_, is zero. The aim is to assess the ability of the algorithm to recover the true distribution *θ*_*k*_ from a set of observed read counts *y*_*k*_, and, more specifically, determine whether spurious patterns can be identified.

The algorithm can be run in two possible modes: a slow (exact) mode, which performs a complete calculation over all possible 2^*n*^ methylation patterns, and an (approximate, default) fast mode which assumes *θ*_*k*_=0 for any pattern for which the observed read count *y*_*k*_ is zero. Because of the exponential growth in the number of patterns, we find in general that use of the function in the slow mode becomes computationally prohibitive for *n*>8. However, there is very little difference in the computed results between the two modes (see below). Furthermore, the number of realised patterns tends to be relatively small and the performance of the function is adequate even for large *n*.

**Figure 1:**
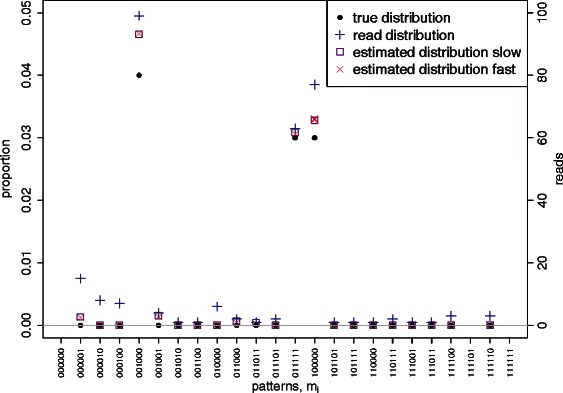
The ‘true’ distribution *θ*_*k*_ (black dots), observed read counts *y*_*k*_ (blue pluses) and estimated distribution ${\widehat{\theta}}_k$ from the slow (purple squares) and fast (red crosses) implementations of our algorithm over methylation patterns for synthetic data with *n*=6 CpG sites. The non-conversion rate is set to be *ε*=0.005, the sequencing errors are set to be *η*=(0.008,0.006,0.006,0.006,0.006,0.008), the total number of reads is *N*_read_=2000. Only patterns with non-zero reads are shown and methylation patterns are ordered lexicographically. Data for the three patterns (000000, 111101 and 111111) which are beyond the range of the plot can be found in Table 1.

To illustrate the concept of the algorithms, we begin with a small synthetic dataset corresponding to a locus with *n*=6 CpG sites, and a distribution of pattern abundances *θ*_*i*_ contrived to mimic a combination of high and low abundance patterns as observed in a real dataset. Table 1 and Figure 1 show the results of creating the dataset assuming a non-conversion rate *ε*=0.005, sequencing errors *η*=(0.008,0.006,0.006,0.006,0.006,0.008) to model a signal that degrades towards the ends of the reads, and a total number of reads *N*_read_=2000 to generate read counts *y*_*i*_. These data are then analysed using the same *ε* and *η* values in both the slow and fast modes to generate estimated pattern abundances ${\widehat{\theta}}_i$.

**Table 1:** **Comparison between estimates** ${\widehat{\theta}}_i$ **of the true methylation distribution** ***θ***_***i***_ **calculated with the slow and fast implementations of our algorithm for the dataset of Figure** 1

**Patterns**	***θ***_***i***_	***y***_***i***_	***y***_***i***_ **/** ***N***_**read**_	${\widehat{\theta}}_i$ ** (slow)**	${\widehat{\theta}}_i$ ** (fast)**
000000	0.50	907	0.4535	0.4813	0.4812
000001	0.00	15	0.0075	0.0013	0.0013
000010	0.00	8	0.0040	0	0
000100	0.00	7	0.0035	0	0
001000	0.04	99	0.0495	0.0466	0.0466
001001	0.00	4	0.0020	0.0016	0.0014
001010	0.00	1	0.0005	0	0
001100	0.00	1	0.0005	0	0
010000	0.00	6	0.0030	0	0
011000	0.00	2	0.0010	0.0007	0.0004
011011	0.00	1	0.0005	0.0005	0.0002
011101	0.00	2	0.0010	0	0
011111	0.03	63	0.0315	0.0308	0.0306
100000	0.03	77	0.0385	0.0328	0.0329
101101	0.00	1	0.0005	0	0
101111	0.00	1	0.0005	0	0
110000	0.00	1	0.0005	0	0.0000
110111	0.00	2	0.0010	0	0
111001	0.00	1	0.0005	0	0
111011	0.00	1	0.0005	0	0
111100	0.00	3	0.0015	0	0
111101	0.20	393	0.1965	0.2001	0.2013
111110	0.00	3	0.0015	0	0
111111	0.20	401	0.2005	0.2044	0.2040

*y*_*i*_ is the number of observed reads for pattern *i* and *N*_read_ is the total number of reads.

For this synthetic dataset we find that for any pattern for which the ‘observed’ read count *y*_*i*_ is zero, the ‘true’ abundance *θ*_*i*_ and the estimated abundance ${\widehat{\theta}}_i$ from the slow-mode version of the algorithm are also zero. For this reason, results are only shown for patterns for which the read count *y*_*k*_ is non-zero. For completeness, the plot produced for all 64 patterns by running our package in the slow mode is given in Additional file 1.

We observe that for almost all patterns the estimated distribution ${\widehat{\theta}}_k$ is closer to the true distribution *θ*_*k*_ than the naive read proportion *y*_*k*_/*N*_read_, the only exception being a slight shift in the wrong direction for the pattern *m*_64_(111111).

We observe also that the algorithm has the effect of skewing the distribution of reads away from low-frequency, more highly methylated patterns towards the totally unmethylated pattern, thus reducing the false discovery rate. This algorithmic effect will generally be the case for any realistic dataset (see Additional file 2). Furthermore, our implementation of the algorithm registers whether, for any given pattern *k*, the estimate ${\widehat{\theta}}_k$ is identically zero, that is, it makes a call as to whether the pattern is present or absent. In this example, no real pattern is classified as spurious. The algorithm correctly identifies 14 out of the 18 spurious patterns in the slow mode, and 13 out of the 18 in the fast mode. For the one extra pattern which was missed by the fast mode, namely pattern 110000, the estimated proportion ${\widehat{\theta}}_i\left(\mathrm{fast}\right)<1{0}^{-4}$ is very low.

In general, when the number of CpG sites *n* in an amplicon is large, it is expected that only a small faction of the 2^*n*^ possible patterns will be present. Furthermore, it is rare for a true pattern with *θ*_*k*_>0 to have zero counts as a result of incomplete bisulphite conversion or sequencing errors. For instance, in the the current example none of the patterns with a positive true frequency *θ*_*k*_ had zero reads, and for the remaining patterns there is no substantial difference between the two implementations of the algorithm. From now on, the discussion focusses on the fast implementation.

**Figure 2:**
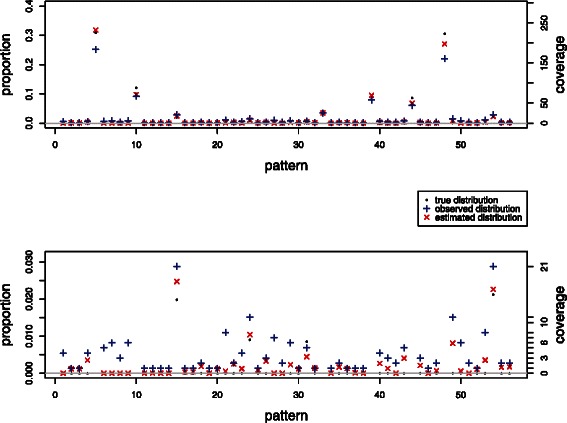
Comparison between the true distribution *θ*_*i*_, the simulated read distribution *y*_*i*_/*N*_read_, and the estimated distribution ${\widehat{\theta}}_i$ for a synthetic dataset based on amplicon data. Parameter values are *n*=9 CpG sites, total number of reads *N*_read_=730, non-conversion parameter *ε*=0.01, and sequencing error rate *η*=0.02. The lower plot is an expanded version of the upper plot showing only patterns with low frequencies.

Figure 2 and Additional file 3 show the results of applying the fast implementation of the algorithm to synthetic data modelled on biological data from a PCR amplicon in the honey bee *Apis mellifera* genome (see Biological data section). To obtain the dataset, the function was applied once to a biological dataset of *N*_read_=730 reads from an amplicon corresponding to a locus with *n*=9 CpG sites. To maintain a similar number of non-zero reads the ‘true’ distribution of the synthetic dataset was taken to be

$$ {\theta}_i=\left\{\begin{array}{ll}0& \kern1em \mathrm{if}\kern6em {\widehat{\theta}}_i^{\mathrm{init}}<0.005,\\ {}{\widehat{\theta}}_i^{\mathrm{init}}\kern5em & \mathrm{otherwise},\end{array}\right. $$

where ${\widehat{\theta}}_i^{\mathrm{init}}$ is the result of the initial application of the algorithm. Here the non-conversion parameter is *ε*=0.01, and the sequencing error rate *η*=0.02 is taken to be uniform across all CpG sites.

Of the 46 spurious patterns in this dataset, 16 are correctly identified and no false identifications of spurious patterns are made. For the 30 failed identifications, the program estimates a lower estimate ${\widehat{\theta}}_i$ than the read proportion *y*_*i*_/*N*_read_.

**Figure 3:**
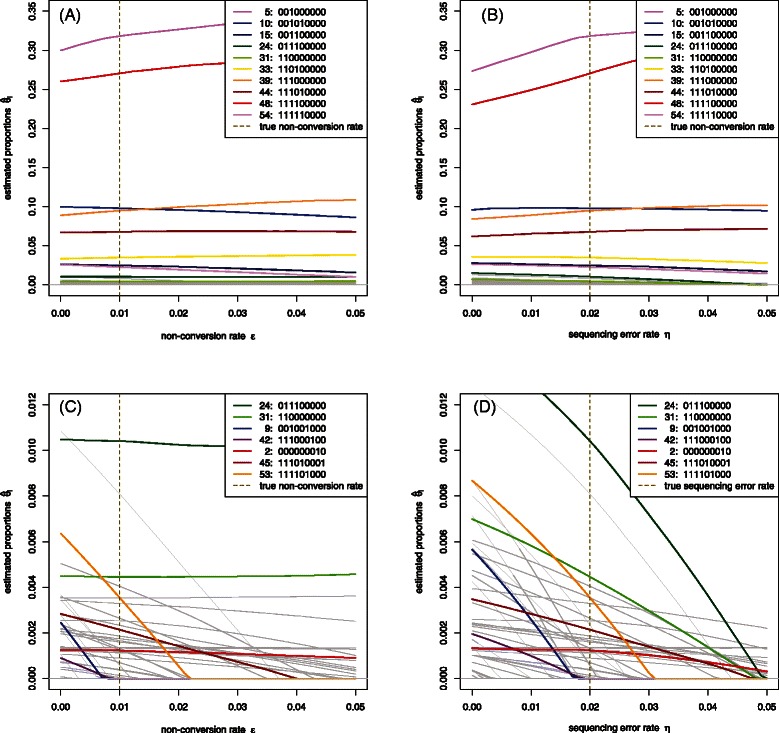
Estimates ${\widehat{\theta}}_i$ of methylation pattern abundances calculated using MPFE from the synthetic dataset of Figure 2 assuming, in panels **(A)** and **(C)**, a range of non-conversion rates *ε* and the correct sequencing error rate *η*=0.02, and, in panels **(B)** and **(D)** the correct non-conversion rate *ε*=0.01 and a range of sequencing error rates *η*. Pattern numbers in the legends correspond to the horizontal axis of Figure 2. In panels (A) and (B) the 10 ‘true’ patterns are highlighted on colour. Panels (C) and (D) show an expanded view of the lower part of the plot with examples of patterns occuring at low frequencies (both spurious and non-spurious) highlighted in colour: 2 real patterns (24 and 31), 3 spurious patterns which are reported as real in Figure 2 (namely 2, 45 and 53), and 2 spurious patterns which are correctly identified in Figure 2 (namely 9 and 42).

Given that the MPFE algorithm requires as input an estimate of the non-conversion rate *ε* and read error rate *η*, it is important to gauge the sensitivity of pattern frequency estimates to these parameters. Figure 3 shows the variation in estimated pattern abundances for the dataset of Figure 1 as the values of *ε* and *η* used by MPFE are perturbed from the ‘true’ values used to construct the synthetic data. We observe that the estimated abundances are relatively insensitive to *ε* for the actually occurring patterns, but drop rapidly with increasing *ε* for most of the spurious patterns. It is perhaps not surprising that ${\widehat{\theta}}_i$ will be sensitive to the *ε* for those spurious patterns for which ${\widehat{\theta}}_i>0$: As explained in Additional file 2, increasing *ε* has the effect of skewing the distribution towards the less methylated patterns, which in general tend to be the more abundantly ocurring patterns. A relevant point here is that the correction for non-conversion almost invariably moves the estimates ${\widehat{\theta}}_i$ in the correct direction from the raw data for the spurious patterns, while leaving even the low-frequency non-spurious patterns almost unchnaged. Also, not surprisingly, the estimates ${\widehat{\theta}}_i$ are sensitive to the read error rate *η* for both spurious and non-spurious patterns.

### Detecting spurious patterns

The effectiveness of our algorithm in identifying spurious patterns can be further gauged by introducing a threshold on the estimated pattern abundance. In the following definitions we only consider methylation patterns with non-zero reads *y*_*i*_.

We set a threshold *K*∈[0,1] as a binary classifier and, using the estimated distribution ${\widehat{\theta}}_i$ as a test statistic, declare pattern *i* to be spurious when ${\widehat{\theta}}_i\le K$. Patterns are defined to be true or false positives or negatives according to the rules

$$ \begin{array}{c}\mathrm{T}\mathrm{P}\ \mathrm{if}\kern2em {\widehat{\theta}}_i\le K\kern2em \mathrm{and}\kern2em {\theta}_i=0,\kern2em \mathrm{F}\mathrm{P}\ \mathrm{if}\kern2em {\widehat{\theta}}_i\le K\kern2em \mathrm{and}\kern2em {\theta}_i>0,\\ {}\mathrm{T}\mathrm{N}\ \mathrm{if}\kern2em {\widehat{\theta}}_i>K\kern2em \mathrm{and}\kern2em {\theta}_i>0,\kern2em \mathrm{F}\mathrm{N}\ \mathrm{if}\kern2em {\widehat{\theta}}_i>K\kern2em \mathrm{and}\kern2em {\theta}_i=0.\end{array} $$

True positive rates (TPR) and false positive rates (FPR) are defined in the usual way as

$$ \mathrm{T}\mathrm{P}\mathrm{R}=\frac{\mathrm{TP}}{\mathrm{TP}+\mathrm{F}\mathrm{N}},\kern2em \mathrm{F}\mathrm{P}\mathrm{R}=\frac{\mathrm{FP}}{\mathrm{FP}+\mathrm{T}\mathrm{N}}. $$

An analogous set of definitions applies using the raw data count proportions *y*_*i*_/*N*_read_ as a test statistic. Note that this classification does not constitute classical hypothesis testing per se, as the threshold does not easily translate to a p-value under a well-defined null hypothesis: patterns are not independent, and specifiying *θ*_*i*_=0 for a given pattern does not determine the distribution of the estimator ${\widehat{\theta}}_i$ without further assumptions about the remaining 2^*n*^−1 patterns.

**Figure 4:**
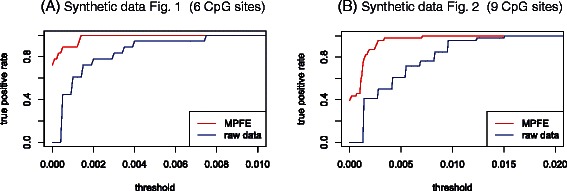
The TPR versus the threshold *K* using the test statistic ${\widehat{\theta}}_i$ (red curve) and *y*_*i*_/*N*_read_ (blue curve) for the synthetic data of **(A)** Figure 1 and **(B)** Figure 2.

Figure 4A shows the TPR curves for the data of Figure 1. It shows that using ${\widehat{\theta}}_i$ results in a clear improvement in detecting which methylation patterns are likely to be a spurious artefact of incomplete conversion and reading error. The FPR curves for both test statistics are constantly zero for the same threshold range in the TPR graph.

Figure 4B shows the TPR curves for the synthetic data based on biological data from an amplicon analysed in Figure 2. Again we observe a clear improvement in detecting which methylation patterns are likely to be a spurious artefact of incomplete methylation and reading error.

### Biological data

Amplicons were obtained as described in [4]. Briefly, genomic DNA was extracted from brains of adult honeybee workers and treated with sodium bisulphite. A region of gene GB17113 (gene ID: 724724, a 6-phosphofructokinase) was then amplified by PCR and sequenced using the 454 technology. No ethical approval was required for this work.

**Figure 5:**
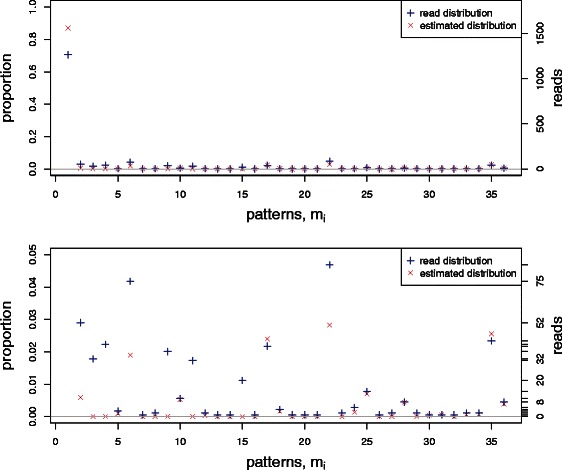
Estimated distribution ${\widehat{\theta}}_i$ of the methylation patterns obtained for a honey bee amplicon. Parameter values are *n*=8 CpG sites, total number of reads *N*_read_=1793, non-conversion rate *ε*=0.01, and sequencing error rate *η*=0.02. The lower plot is an expanded version of the upper plot showing only patterns with low frequencies. The 36 patterns with non-zero reads are listed in Table 2.

Below we apply the fast algorithm to two examples from this dataset assuming a non-conversion rate of *ε*=0.01 and a global sequencing error rate of *η*=0.02. The first example is shown in Figure 5 and Table 2. The parameter values are *n*=8 CpG sites, 36 patterns with non-zero reads, and a total number of reads *N*_read_=1793.

**Table 2:** **Estimated methylation distribution** ${\widehat{\theta}}_i$ **for the data of Figure** 5

***i***	***m***_***i***_	***y***_***i***_	***y***_***i***_ **/** ***N***_**read**_	${\widehat{\theta}}_i$
1	00000000	1265	0.7055	0.8706
2	00000001	52	0.0290	0.0059
3	00000010	32	0.0178	0
4	00000100	40	0.0223	0
5	00000110	3	0.0017	0.0010
6	00001000	75	0.0418	0.0189
7	00001001	1	0.0006	0
8	00001010	2	0.0011	0
9	00010000	36	0.0201	0
10	00010100	10	0.0056	0.0053
11	00100000	31	0.0173	0
12	00100001	2	0.0011	0.0003
13	00101000	1	0.0006	0
14	00110000	1	0.0006	0
15	01000000	20	0.0112	0
16	01000001	1	0.0006	0
17	01100000	39	0.0218	0.0240
18	01100001	4	0.0022	0.0016
19	01101000	1	0.0006	0
20	01110000	1	0.0006	0
21	01110100	1	0.0006	0
22	10000000	84	0.0468	0.0282
23	10000001	2	0.0011	0
24	10000010	5	0.0028	0.0013
25	10000100	14	0.0078	0.0070
26	10001000	1	0.0006	0
27	10010000	2	0.0011	0
28	10010100	8	0.0045	0.0044
29	10100000	2	0.0011	0
30	11000100	1	0.0006	0.0003
31	11010000	1	0.0006	0.0005
32	11100000	1	0.0006	0
33	11100100	2	0.0011	0.0006
34	11110000	2	0.0011	0.0007
35	11110100	42	0.0234	0.0255
36	11110110	8	0.0045	0.0039

The 36 patterns with non-zero reads are labelled *m*_*i*_, *i*=1,…,36 and *y*_*i*_ are the observed read counts.

There are several observations:

(i) 18 patterns (50%) are identified as spurious;
(ii) there are 11 patterns with only 1 read - our algorithm calls 9 of them as spurious, while predicts the other 2 patterns (*m*_30_ and *m*_31_) to exist;
(iii) patterns *m*_3_, *m*_4_, *m*_9_ and *m*_11_ with >30 reads each has a read proportion *y*_*i*_/*N*_read_≈2*%*, but are called as spurious.

Observations (i) and (ii) can be explained by the fact that the edit distance between the two patterns covered by a single read and any pattern observed to be highly abundant renders it unlikely that these patterns have arisen through sequencing errors or incomplete conversion. Observation (iii) arises because the spurious patterns with >30 reads are just one sequencing error or one incomplete conversion away from the most abundant pattern, *m*_0_(00000000).

**Figure 6:**
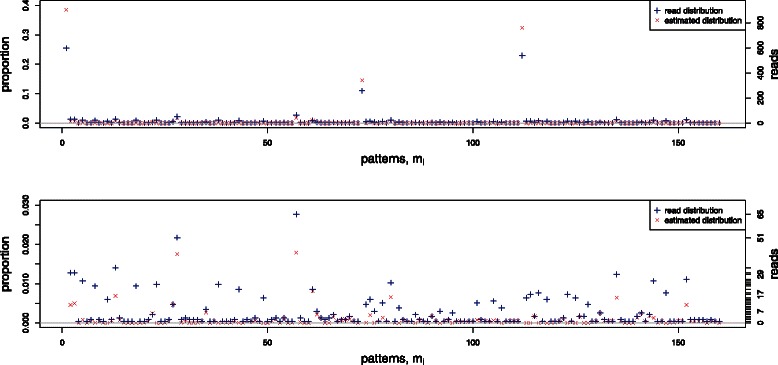
Estimated distribution ${\widehat{\theta}}_i$ of the methylation patterns for a second amplicon from the honey bee genome. Parameter values are *n*=14 CpG sites, total number of reads *N*_read_=2347, non-conversion rate *ε*=0.01, and sequencing error rate *η*=0.02. The lower plot is an expanded version of the upper plot showing only patterns with low frequencies.

Figure 6 and Additional file 4 show the second example, with *n*=14 CpG sites and 160 patterns with non-zero reads. The total number of reads is *N*_read_=2347. In this case 47 patterns are called as spurious. Note that these data indicate that the methylation statuses of neighouring CpG loci are correlated. This correlation was also observed by Lyco et al. [4] and has motivated our approach that has the advantage of being able to accommodate neighbouring spatial correlations between CpG positions without imposing any a priori structure.

## Conclusions

We have developed an algorithm for estimating the true distribution of methylation patterns in at a genomic locus containing *n* CpG sites. The algorithm, based on a constrained multinomial model, accounts for statistical variation due to incomplete bisulphite conversion and sequencing errors. This method can be readily applied to methylation patterns extracted from amplicon sequencing data analysed with with software such as BiQ-Analyser [17]. Further work would be required to extract methylation patterns counts from whole-genome sequencing data.

The analysis differs from previous treatments in that the estimated distribution is a joint probability distribution over patterns which preserves maximal information pertaining to interaction between different CpG sites, as opposed to a pointwise measure of methylation at each site. A pointwise methylation estimate can, of course, be recovered from our estimated distribution as a marginal distribution. The algorithm is implemented as the R Bioconductor package MPFE.

Numerical experiments with realistic synthetic data indicate that the algorithm is able to identify the majority of the spurious observed methylation patterns, that is, patterns which are not present in the original library but are observed in the reads because of incomplete bisulphite conversion or sequencing errors. In general, our estimates are closer to the true distribution than the naive estimates given by the relative proportion of observed read counts for almost all patterns in each simulation (see Figures 1, 2, and Table 1).

Application of the algorithm to biological data consisting of bisulphite treated amplicon reads for a honeybee genomic sequence predicts that a correspondingly high proportion of observed methylation patterns in real data may indeed be spurious. However, our results also reveal an important number of real methylation patterns in this biological sample. This complexity of the methylation landscape is virtually undetectable when one only considers position-wise methylation level, but becomes apparent through our method.

In the future, the statistical method presented here could be extended and refined to deal with systematic sources of errors (for instance non-homogeneous bisulfite conversion rates, differential amplification of different epialleles) and to extract information from incomplete data (reads where one or more positions of the pattern are missing, which are currently ignored).

## Methods

### Statistical model of bisulphite sequencing

We take as the starting point of our statistical model a population of methylomes restricted to a locus containing *n* CpG sites. Each member of the population within a given class is represented by a vector of non-independent binary valued random variables **K**=(*K*_1_,…,*K*_*n*_), where each *K*_*s*_∈{0,1} labels the methylation state (0 for unmethylated, 1 for methylated) at the *s*-th CpG site at this locus. The population defines a methylation profile represented by the probability distribution of realising the pattern **k**=(*k*_1_,…,*k*_*n*_) in a read randomly chosen from the population:

(1) $$ {\theta}_{\mathbf{k}}= Prob\kern0.3em \left(K=k\right),\kern1em k\in {\left\{0,1\right\}}^n. $$

For convenience, from here on we will label the possible methylation patterns by the integers *k*=1,…,2^*n*^, and set *θ*_**k**_=*θ*_*k*_, where *k*−1 is the integer whose binary representation is the methylation pattern **k**.

Our aim is to estimate the distribution *θ*_*k*_ representing the relative abundance of methylation pattern *k* from high throughput sequencing data consisting of a set of integer valued read counts *Y*_*k*_. In a typical experiment, the number *n* of CpG sites in an amplicon may be up *O*(10^2^), and the total number of read counts ${N}_{\mathrm{read}}=\sum_{k=1}^{2^n}{Y}_k$ may be up to *O*(10^6^).

The model takes into account two sources of error. First, the bisulphite conversion of unmethylated cytosine to uracil is not 100% efficient. There is a probability *ε* that an unmethylated CpG site will register as being methylated, where *ε*∼*O*(10^−2^) can be estimated from the cytosines known not to be methylated (mitochondrial genome, chloroplastic genome, spike-ins, etc). The second type of error is caused by sequencing. For many applications this may be assumed for practical purposes to be site independent. However, to allow for effects such as degradation of the read quality towards the ends of the reads, we will assume there is a site-dependent probability *η*_*s*_∼*O*(10^−2^) that if site *s* is unmethylated it will register as methylated and vice versa. It follows that if the true methylation pattern of any given read is **K**, but the read registers as being pattern **L**, then

(2) $$ Prob\kern0.3em \left(L=\ell \Big|K=k\right)={M}_{k\ell },\kern1em k,\ell =1,\dots {2}^n, $$

where, as above, we adopt the conventon that *k*−1 is the integer whose binary representation is the methylation pattern **k**=(*k*_1_,…,*k*_*n*_) and *ℓ*−1 is the integer whose binary representation is the methylation pattern **l**=(*ℓ*_1_,…,*ℓ*_*n*_), and

(3) $$ {M}_{k\ell }={\left({E}_1\right)}_{k_1{\ell}_1}{\left({E}_2\right)}_{k_2{\ell}_2}\dots {\left({E}_n\right)}_{k_n{\ell}_n}, $$

where, for each *s*=1,…*n*,

(4) $$ \begin{array}{lcrr}{E}_s& =& \left(\begin{array}{ll}1-\varepsilon & \varepsilon \\ {}0& 1\end{array}\right)\left(\begin{array}{ll}1-{\eta}_s& {\eta}_s\\ {}{\eta}_s& 1-{\eta}_s\end{array}\right)& \\ {}& =& \left(\begin{array}{ll}1-\varepsilon -{\eta}_s+2\varepsilon {\eta}_s& \varepsilon +{\eta}_s-2\varepsilon {\eta}_s\\ {}{\eta}_s& 1-{\eta}_s\end{array}\right).& \end{array} $$

is a 2 ×2 matrix whose rows and columns are labelled 0 (for unmethylated) and 1 (for methylated). In fact the matrix *M* whose elements are *M*_*kℓ*_ is the Kronecker product *E*_1_⊗…⊗*E*_*n*_.

Eqs. (1) and (2) imply that the probability that a random read will be the pattern *ℓ* is

(5) $$ Prob\kern0.3em \left(L=\ell \right)=\sum_{k=1}^{2^n}{\theta}_k{M}_{k\ell }={\phi}_{\ell }, $$

say. Assuming each read to be independent, for an experiment with a given total number of reads *N*_read_ the observed set of read counts represented by the random variable *Y*_*ℓ*_ has a multinomial distribution:

(6) $$ Prob\kern0.3em \left({Y}_{\ell }={y}_{\ell}\Big|\phi \right)=\frac{N_{\mathrm{read}}!}{y_1!{y}_2!\dots {y}_{2^n}!}{\phi_1}^{y_1}\dots {\phi_{2^n}}^{y_{2^n}}. $$

In a recent applications note, [9] develop a statistical model for the distribution of the number of reads which register as being methylated in a pooled set of bisulphite-sequencing reads from CpG sites in a given region of a genome. Their model is mathematically equivalent to the *n*=1 version of the above model, and as such can be simplified to a single binomial distribution (see Additional file 2).

### Parameter estimation

The parameters of the distribution over methylation profiles, *θ*_*k*_, are estimated by maximising the log likelihood:

(7) $$ L\left(\theta \Big|y\right)=\sum_{k=1}^{2^n}{y}_k \log \left(\sum_{j=1}^{2^n}{\theta}_j{M}_{jk}\right), $$

subject to the constraint that $\left({\theta}_1,\dots, {\theta}_{2^n}\right)$ lies in the (2^*n*^−1)-dimensional simplex

(8) $$ S=\left\{\theta :\sum_{k=1}^{2^n}{\theta}_k=1,{\theta}_k\ge 0\right\}. $$

**Figure 7:**
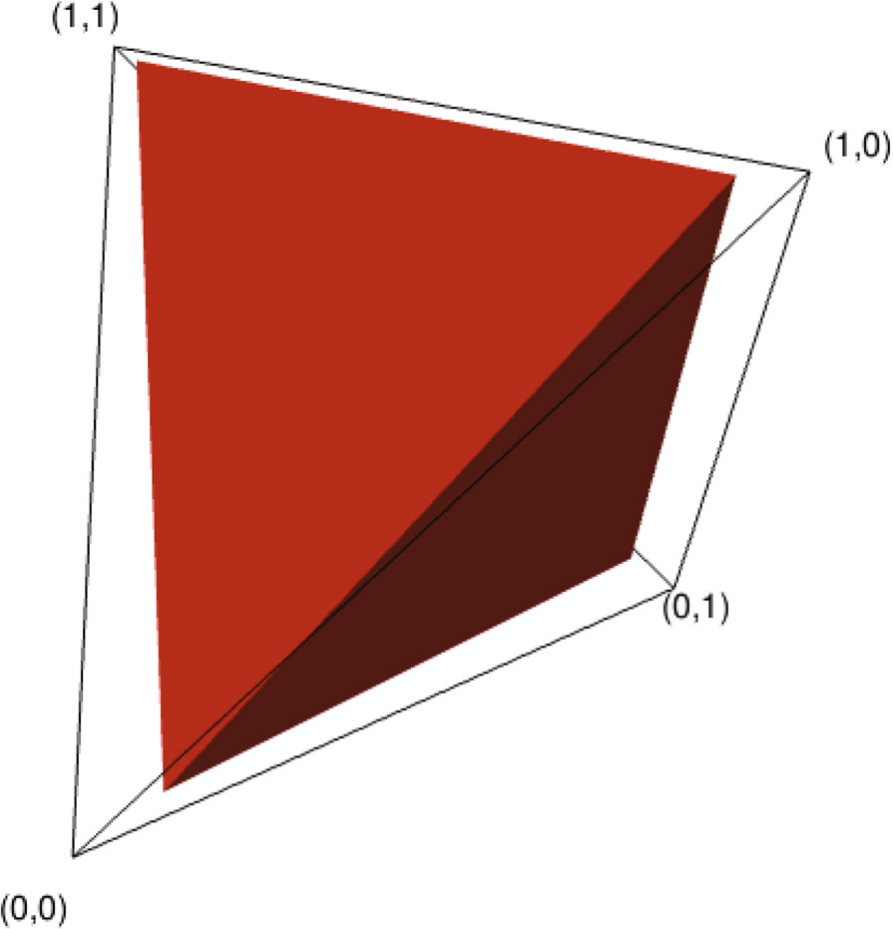
Boundary of the allowed simplex Eq. (8) for the parameters *θ*, (black wire frame) and corresponding shrunken simplex containing allowed values of *ϕ* (red tetrahedron) for the case *n*=2 CpG sites, *ε*=0.05, *η*_*s*_=0.02. Numbers at the corners are the 2^*n*^=4 possible methylation patterns).

One may be tempted to use the usual formula, ${\widehat{\phi}}_{\ell }={y}_{\ell }/{N}_{read}$, for the maximum likelihood estimate of multinomial parameters, and simply invert the matrix *M* to recover ${\widehat{\theta}}_k$. However this will not work for any realistic data because the matrix *M* shrinks the simplex to a smaller volume. In practice many of the *y*_*ℓ*_ are zero, which leads to a naive estimate ${\widehat{\phi}}_{\ell }$ on the boundary of the unshrunken simplex in *ϕ*-space, and this boundary is not included in the shrunken simplex (see Figure 7 for the *n*=2 case, in which the simplex is tetrahedron).

Instead we maximise the log likelihood over the allowed domain numerically by using the R function constrOptim(). Unfortunately the performance of this function becomes prohibitively slow for $n\gtrsim 8$ as the dimensionality of the parameter space grows exponentially. However, we have noticed in numerical simulations that if the observed counts *y*_*k*_ are zero for a subset of the possible patterns, the corresponding estimates ${\widehat{\theta}}_k$ are zero (or in rare cases, very close to zero) for the same subset. Thus we have implemented an algorithm which only searches over that part of the boundary of *S* constrained by *θ*_*k*_=0 for those *k* such that *y*_*k*_=0. The algorithm remains reasonably efficient on a standard desktop computer provided the number of observed methylation patterns does not exceed about 200. This allows analysis of most realistic datasets while still addressing the biologically relevant question of identifying spurious methylation patterns which are the result of incomplete methylation.

In Additional file 2 we prove that both the exact maximum likelihood estimate, and the aproximate maximum likelihood estimate over reduced part of the boundary described above are unique. However, in both cases the function constrOptim() is of finite accuracy in locating the maximum of the log-likelihood. Thus, if the located maximum $\widehat{\theta}$ is close to the boundary of the simplex, our algorithm also calculates the value of the log-likelihood at several nearby points on the boundary. If this results in a log-likelihood bigger than or equal to the maximum reported by constrOptim(), the appropriate point on the boundary is taken as the maximum likelihood estimate, and those patterns *m*_*k*_ for which ${\widehat{\theta}}_k\equiv 0$ are reported as being spurious reads.

Additional file 2 also contains an argument that for realistic datasets and parameter values, the estimated distribution ${\widehat{\theta}}_i$ is in general skewed towards less-methlyated states relative to the naïve estimate *y*_*i*_/*n*_read_, and that the set of patterns reported to be present is a subset of (or the same set as) the set of patterns naïvely observed. These observations hold rigorously for any dataset if the algorithm is run with the read-error rate *η*_*s*_ set to zero for all sites *s*.

## Additional files

Additional file 1
**Estimated pattern abundances using the exact algorithm.** Estimates ${\widehat{\theta}}_i$ calculated with the exact, slow implementations of our algorithm for the synthetic dataset of Figure 1. Methylation patterns are labelled lexicographically from *m*_1_=000000 to *m*_64_=111111. Data for patterns *m*_1_, *m*_62_=111101, and *m*_64_ are beyond the range of the plot, but are listed in Table 1.

Additional file 2
**Technical details.** Analysis of the relationship of the statistical model used by Akman et al. [9] to our statistical model, proof of uniqueness of maximum likelihood estimate and argument that the number of reported methylation patterns is reduced by the MPFE algorithm.

Additional file 3
**Table of results for the second synthetic data example.** True and estimated methylation distributions *θ*_*i*_ and ${\widehat{\theta}}_i$ for the data of Figure 2. The 56 patterns with non-zero reads are labelled by their binary representation and *y*_*i*_ are the observed read counts.

Additional file 4
**Table of results for the second biological data example.** Estimated methylation distribution ${\widehat{\boldsymbol{\theta}}}_{\boldsymbol{i}}$ for the data of Figure 6. The 160 patterns with non-zero reads are labelled by their binary reresentation and *y*_*i*_ are the observed read counts.

## Abbreviations

CpG: Cytosine-phosphate-guanine dinucleotide
DNA: Deoxyribonucleic acid
FN: False negative
FP: False positive
FPR: False positive rate
PCR: Polymerase chain reaction
TN: True negative
TpG: Thymine-phosphate-guanine dinucleotide
TPR: True positive rate
