# Supplementary figures and images for: Estimation of the methylation pattern distribution from deep sequencing data
Source: BMC Bioinformatics. 2015 May 6;16:145. doi: 10.1186/s12859-015-0600-6 (PMC4428226; doi:10.1186/s12859-015-0600-6)

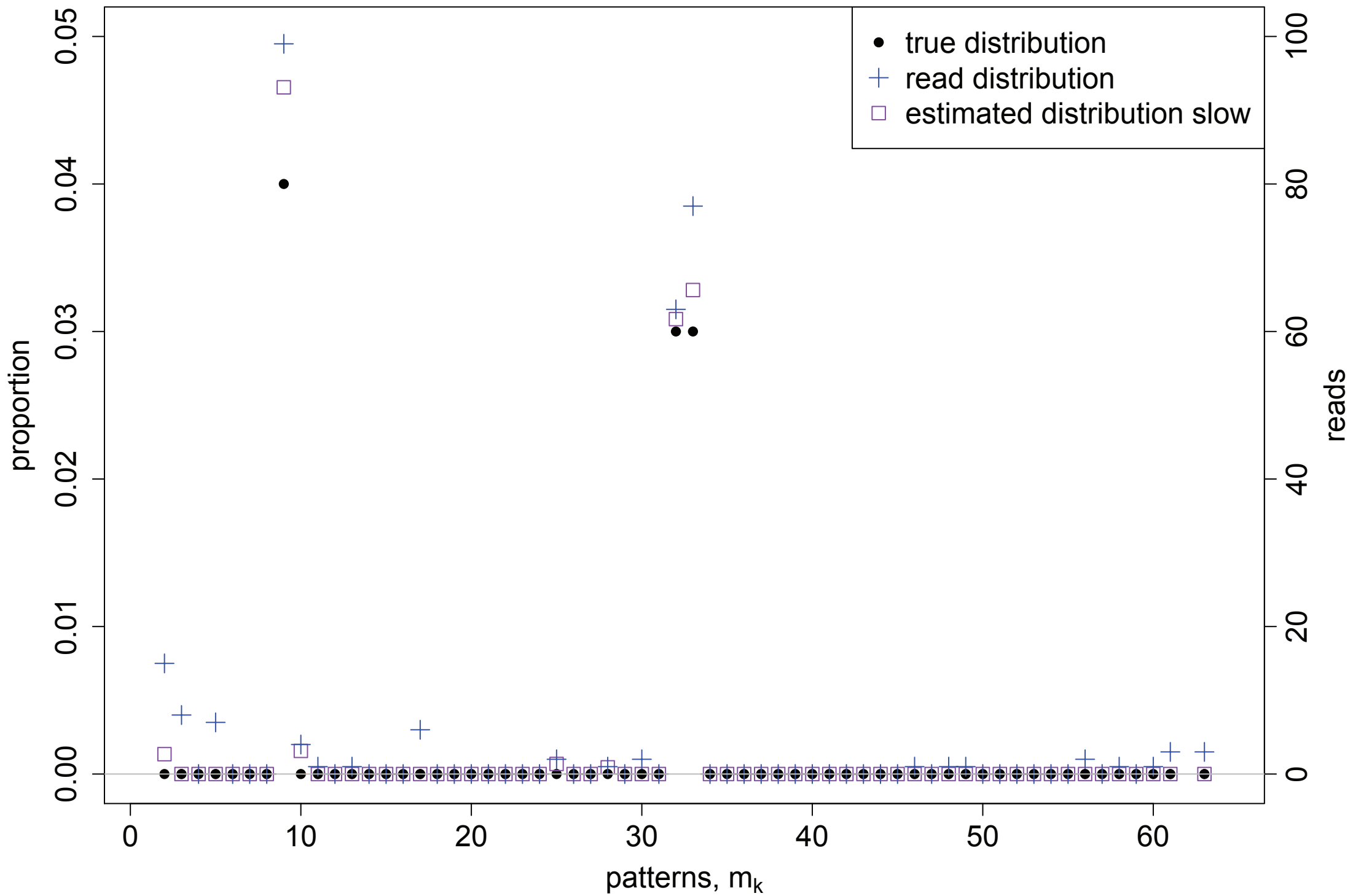

Supplement: Additional file 1 — Estimated pattern abundances using the exact algorithm. Estimates ${\widehat{\theta}}_i$θ^i calculated with the exact, slow implementations of our algorithm for the synthetic dataset of Figure 1. Methylation patterns are labelled lexicographically from m 1=000000 to m 64=111111. Data for patterns m 1, m 62=111101, and m 64 are beyond the range of the plot, but are listed in Table 1. [file 12859_2015_600_MOESM1_ESM.pdf]
