# Supplementary material for: Estimation of the methylation pattern distribution from deep sequencing data
Source: BMC Bioinformatics. 2015 May 6;16:145. doi: 10.1186/s12859-015-0600-6 (PMC4428226; doi:10.1186/s12859-015-0600-6)
Supplement: Additional file 2 — Technical details. Analysis of the relationship of the statistical model used by Akman et al. [9] to our statistical model, proof of uniqueness of maximum likelihood estimate and argument that the number of reported methylation patterns is reduced by the MPFE algorithm. [file 12859_2015_600_MOESM2_ESM.pdf]

Estimation of amplicon methylation patterns from bisulphite sequencing data
Additional File 2: Technical details

Peijie Lin, Sylvain Forêt, Susan R. Wilson and Conrad J. Burden

February 25, 2015

Comparison with Akman et al. (2014)

Akman et al. [1] have developed the Bioconductor package BEAT (BS-Seq Epimutation Analysis Toolkit) for quantitative analysis of DNA methylation patterns from bisulphite sequencing data. The basis of their software is a statistical model for the distribution of the number of reads which register as being methylated from a pooled set of bisulphite-sequencing reads from CpG sites in a given region of a genome. The distribution is derived in terms of the total number of pooled reads from the region, a false positive rate and a false negative rate representing incorrect conversions or non-conversions, and an underlying methylation rate which is assumed to be common to all CpG sites within the region. Their purpose is to estimate the common underlying methylation rate for the region. Their model does not deal with the distribution over methylation patterns.

We argue that because of the assumption that all CpG sites within the pooled region share common values for the distributional parameters, their model is mathematically equivalent to the $n = 1$ CpG site version of the model set out in Section 3.1 of the Methods section of the current paper. More importantly, the binomial mixture model arrived at by Akman et al. by repeated application of the law of total probability is nothing more than a single binomial distribution. Once this is recognised, it is a trivial matter to estimate the underlying methylation rate from maximum likelihood without the need for the Bayesian analysis employed by Akman et al.

Table 1 lists the correspondence between the notation of Akman et al. and that used the current paper. If we represent the number of of reads registering as being methylated by the random variable K, then Eq. (3) of the supplementary material of Akman et al. states that

$$K \sim \sum_{j=0}^{n} \sum_{m=0}^{k} \mathrm{Bin}(m; j, 1 - p_-)\mathrm{Bin}(k - m; n - j, p_+)\mathrm{Bin}(j; n, r).$$

	Akman et al.	Lin et al.
Number of CpG reads	n	N_{read}
Number of reads registering as methylated	k	y
Underlying methylation rate	r	θ
False positive rate	p_+	$\epsilon + \eta - 2\epsilon\eta$
False negative rate	p_-	η

Table 1: Correspondence between the notation of Akman et al. and that used in the current paper. The rates p_+ and p_- are the off-diagonal elements of the matrix Eq. (4) in the main paper.

From Eqs. (6) and (5) of the current paper we claim that this is equivalent to single binomial distribution

$$K \sim \mathrm{Bin}(k; n, \phi),$$

where

$$\phi = r + p_+ - r(p_+ + p_-).$$

The equivalence is demonstrated numerically for a specific case by the following R code:

```
> n <- 20
> r <- 0.3
> pPlus <- 0.1
> pMinus <- 0.2
> #
> probAkman <- array(dim=n+1)
> for(k in 0:n){
+   probA <- 0
+         for(j in 0:n){
+           for(m in 0:k){
+                 probA <- probA +
+                   dbinom(m, size=j, prob=1-pMinus)*
+                   dbinom(k-m, size=n-j, prob=pPlus)*
+                   dbinom(j, size=n, prob=r)
+                 }
+           }
+           probAkman[k+1] <- probA
+         }
> #
> phi <- r + pPlus - r*(pPlus + pMinus)
> probLin <- dbinom(0:n, size=n, prob=phi)
> #
> print(data.frame(Akman=probAkman, Lin=probLin,
+           relDifference=2*(probAkman - probLin)/
+                    (probAkman + probLin), row.names=0:n))
```

	Akman	Lin	relDifference
0	5.983865e-04	5.983865e-04	9.059380e-16
1	5.376807e-03	5.376807e-03	8.065770e-16
2	2.294883e-02	2.294883e-02	-6.047274e-16
3	6.186207e-02	6.186207e-02	-5.608359e-16
4	1.181207e-01	1.181207e-01	2.349764e-16
5	1.698199e-01	1.698199e-01	-4.903237e-16
6	1.907397e-01	1.907397e-01	4.365463e-16
7	1.713893e-01	1.713893e-01	-3.238892e-16
8	1.251266e-01	1.251266e-01	4.436398e-16
9	7.495509e-02	7.495509e-02	1.851480e-15
10	3.704302e-02	3.704302e-02	7.492795e-16
11	1.512956e-02	1.512956e-02	-4.586316e-16
12	5.098004e-03	5.098004e-03	-2.722200e-15
13	1.409482e-03	1.409482e-03	-9.230646e-16
14	3.166227e-04	3.166227e-04	-1.883350e-15
15	5.690031e-05	5.690031e-05	-8.336307e-16
16	7.988721e-06	7.988721e-06	-1.272343e-15
17	8.445025e-07	8.445025e-07	-3.510487e-15
18	6.323569e-08	6.323569e-08	-1.674357e-15
19	2.990551e-09	2.990551e-09	-6.914951e-15
20	6.717905e-11	6.717905e-11	-1.154351e-15

Uniqueness of the maximum likelihood estimate of $\theta_1, \ldots \theta_{2^n}$

We demonstrate the uniqueness of the maximum likelihood estimator

$$\hat{\theta} = \underset{\theta_1,\ldots,\theta_{2^n}}{\operatorname{argmax}} \sum_{k=1}^{2^n} y_k \log \left(\sum_{j=1}^{2^n} \theta_j M_{jk} \right), \tag{1}$$

subject to the constraints

$$\sum_{k=1}^{2^n} \hat{\theta}_k = 1, \qquad \hat{\theta}_k \geq 0, \tag{2}$$

where $y_1, \ldots, y_{2^n}$ are the observed read counts and the matrix M is defined by Equation (3) of the main paper. An equivalent formulation of this problem is to determine

$$\hat{\theta}_k = \sum_{j=1}^{2^n} \hat{\phi}_j (M^{-1})_{jk}, \tag{3}$$

where $\hat{\phi}$ is the argmax of the likelihood function

$$L(\phi) = \sum_{k=1}^{2^n} y_k \log \phi_k, \tag{4}$$

subject to the constraints

$$\sum_{k=1}^{2^n} \hat{\phi}_k = 1, \tag{5}$$

which follows from Eq. (2) because M has the properties of a Markov transition matrix, and

$$\sum_{j=1}^{2^n} \hat{\phi}_j (M^{-1})_{jk} \geq 0, \qquad k = 1, \ldots, 2^n. \tag{6}$$

Note that M^{-1} exists provided $\epsilon \neq 1$ and $\eta_s \neq \frac{1}{2}$ (see Eq. (4) of the main paper), which will hold in any realistic situation. An illustrated example of the constraint on $\hat{\phi}$ for the case $n = 2$ is shown in Figure 8 of the main paper.

Suppose, without loss of generality, that the observed read counts are such that

$$y_1, \ldots, y_l > 0, \qquad y_{l+1}, \ldots, y_{2^n} = 0, \tag{7}$$

for some $0 \leq l \leq 2^n$. Then $L(\phi)$ is independent of $\phi_{l+1}, \ldots, \phi_{2^n}$ and

$$\frac{\partial^2 L}{\partial \phi_j \partial \phi_k} = -\delta_{jk} \frac{y_j}{\phi_j^2} \tag{8}$$

is a diagonal matrix whose first l diagonal elements are strictly negative, implying that $L(\phi)$ is a strictly concave function of $\phi_1, \ldots, \phi_l$. Furthermore the set

$$S = \{\phi_1, \ldots, \phi_l | \text{Eqs. (5) and (6) are satisfied}\} \tag{9}$$

is easily shown to be convex, i.e. if $u, v \in S$, then $\lambda u + (1 - \lambda) v \in S$ for all $0 \leq \lambda \leq 1$. It follows that

$$(\hat{\phi}_1, \ldots \hat{\phi}_l) = \underset{(\phi_1, \ldots, \phi_l) \in S}{\arg\max} L(\phi) \tag{10}$$

is unique. Furthermore, $\hat{\phi} = (\hat{\phi}_1, \ldots, \hat{\phi}_{2^n})$ for any choice of $(\hat{\phi}_{l+1}, \ldots, \hat{\phi}_{2^n})$ will also achieve the maximum of $L(\phi)$, but will not in general satisfy the constraints of Eqs. (5) and (6).

Now note that the constraint Eq. (6) together with the fact that the $M_{ki} \geq 0$ implies that

$$\hat{\phi}_i = \sum_{j,k=1}^{2^n} \hat{\phi}_j (M^{-1})_{jk} M_{ki} \geq 0, \quad i = 1, \ldots 2^n, \tag{11}$$

which, together with Eq. (5), implies that

$$\sum_{k=l+1}^{2^n} \hat{\phi}_k = c, \quad \sum_{k=1}^{l} \hat{\phi}_k = 1 - c, \tag{12}$$

for some $0 \leq c \leq 1$. Suppose $c > 0$. Then since $L(\phi)$ is independent of $\phi_{l+1}, \ldots, \phi_{2^n}$ and $\log \phi_k$ is an increasing function of ϕ_k, Eq. (4) can be increased

by simultaneously incrementing $\hat{\phi}_j \to \hat{\phi}_j + \epsilon$ for some $j \leq l$ and $\hat{\phi}_k \to \hat{\phi}_k - \epsilon$ for some $k > l$ such that $\phi_k > 0$, which contradicts Eq (10). Thus we must have $c = 0$, and hence

$$\hat{\phi} = (\hat{\phi}_1, \ldots, \hat{\phi}_l, 0 \ldots, 0) \tag{13}$$

as the unique maximum of Eq. (4) satisfying Eqs. (5) and (6), from which the unique $\hat{\theta}$ is recovered using Eq. (3).

The above argument applies to the exact "slow algorithm" referred to in the main paper. Most of the results reported are obtained with an approximate "fast algorithm", which uses a truncated $l \times l$ submatrix whose elements $\tilde{M}_{jk}$ are equal to M_{jk} for $j, k = 1, \ldots l$. An equivalent formulation of the fast algorithm is to determine

$$\hat{\theta}_k = \sum_{j=1}^{l} \hat{\phi}_j (\tilde{M}^{-1})_{jk}, \tag{14}$$

where $\hat{\phi}$ is the argmax of the likelihood function

$$\tilde{L}(\phi) = \sum_{k=1}^{l} y_k \log \phi_k, \tag{15}$$

subject to the constraints

$$\sum_{j,k=1}^{l} \hat{\phi}_j (\tilde{M}^{-1})_{jk} = 1, \tag{16}$$

and

$$\sum_{j=1}^{l} \hat{\phi}_j (\tilde{M}^{-1})_{jk} \geq 0, \qquad k = 1, \ldots, l. \tag{17}$$

The computational efficiency arises because the number of constraints reduces considerably for large n and physically realistic values of l.

It is easy to check that $\partial^2 \tilde{L}(\phi)/\partial\phi_j \partial\phi_k$ is a diagonal matrix with strictly negative diagonal elements, implying that $\tilde{L}(\phi)$ is a concave function, and that the constraints Eq. (16) and (17) define a convex set. Thus the fast algorithm is also guaranteed to give a unique result.

Reduction in the number of patterns present

One may be tempted to ask whether the maximum likelihood estimate described above is guaranteed to lead to a reduction in the number of methylation patterns reported relative to the number of patterns naïvely observed. We argue here that if read errors are ignored (i.e. we set $\eta = 0$), the estimated distribution over patterns $\hat{\theta}_i$ is shifted, relative to the naïve distribution y_i/n_{read}, towards the totally unmethylated pattern $(0, \ldots, 0)$. As a consequence the set of patterns

reported to be present is guaranteed to be a subset of or equal to the set of observed patterns.

The result is straightforward for the case of $n = 1$ CpG site. In this case we have two patterns: unmethylated (0), and methylated (1). The constraints Eq. (5) and Eq. (6) are

$$\hat{\phi}_{(0)} + \hat{\phi}_{(1)} = 1 \qquad (\hat{\phi}_{(0)}, \hat{\phi}_{(1)}) \begin{pmatrix} 1 & -\epsilon \\ 0 & 1-\epsilon \end{pmatrix} \geq 0, \tag{18}$$

and hence the maximum likelihood estimate is, from Eq. (4),

$$\hat{\phi}_{(0)} = \underset{0 \leq \phi_{(0)} \leq 1-\epsilon}{\operatorname{argmax}} \{y_{(0)} \log \phi_{(0)} + y_{(1)} \log(1 - \phi_{(0)})\}, \quad \hat{\phi}_{(1)} = 1 - \hat{\phi}_{(0)}. \tag{19}$$

The region over which $(\hat{\phi}_{(0)}, \hat{\phi}_{(1)})$ is constrained and its transformation back to the reported abundances $(\hat{\theta}_{(0)}, \hat{\theta}_{(1)})$ are illustrated in Figure 1A.

If the maximum of the function in chain brackets in Eq (19), namely $y_{(0)}/n_{\mathrm{read}}$, lies in the interval $[0, 1-\epsilon]$, the transformation M^{-1} back to θ-space clearly skews the estimated abundances towards the unmethylated state. If the unconstrained maximum lies in the interval $[\epsilon, 1]$, the constrained maximum returns the value $\hat{\phi}_{(0)} = 1 - \epsilon$, which transforms to the estimate $\hat{\theta}_{(0)} = 1$, and any methylated reads are interpreted as spurious. More specifically, the reported estimate is

$$(\hat{\theta}_{(0)}, \hat{\theta}_{(1)}) = \begin{cases} \frac{1}{1-\epsilon}\left(\frac{y_{(0)}}{n_{\mathrm{read}}}, \frac{y_{(1)}}{n_{\mathrm{read}}} - \epsilon\right) & \text{if } \frac{y_{(0)}}{n_{\mathrm{read}}} \leq 1 - \epsilon \\ (1, 0) & \text{if } 1 - \epsilon < \frac{y_{(0)}}{n_{\mathrm{read}}} \leq 1. \end{cases} \tag{20}$$

To see how the result generalises to higher numbers of CpG sites, consider the $n = 2$ case illustrated in Figure 1B. The fully methylated pattern $(1, 1)$ is a fixed point of the transformation M. Points in each triangular face of the black wire tetrahedron correspond to reported distributions in θ-space for which the pattern i indicated at the opposite corner occurs with probability $\hat{\theta}_i = 0$. Thus the transformation M^{-1} of the red tetrahedron to the black wire frame skews the distribution towards less-methylated patterns. Furthermore, whenever the maximum y_i/n_{read} of the likelihood function Eq (4) lies outside the red tetrahedron but inside the wire frame, the constrained maximum lies on the surface of the red tetrahedron, which maps to a point in one of the triangular faces of the wire tetrahedron, and possibly to an edge or corner. In this case at least one pattern is reported to be spurious[1].

For an arbitrary number n of CpG sites, the only fixed point of the $2^n \times 2^n$ matrix M is vector $(1, \ldots, 1)$ corresponding to the fully methylated pattern. Again the transformation M^{-1} skews the distribution towards less-methylated patterns, and any observed pattern with a small number of observed counts can potentially be reported as spurious.

[1]Note that in the extreme, unphysical limit $\epsilon \to 1$, the red tetrahedron shrinks to the corner $(1, 1)$ and all observed patterns except the fully unmmethylated pattern $(0, 0)$ become "spurious".

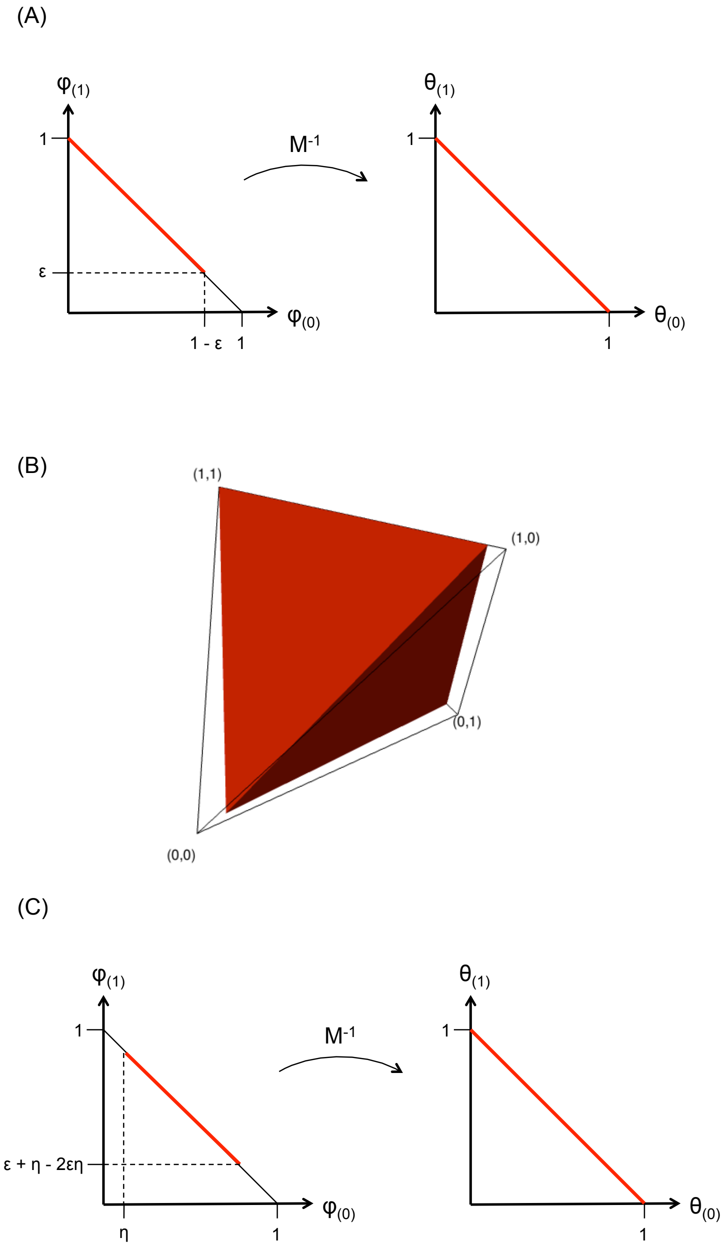

Figure 1: (A) Schematic of the region of ϕ-space constrained by Eq. (5) and Eq. (6) shown in red and its mapping back to θ-space for the case of an amplicon with $n = 1$ CpG site and read-error rate $\eta = 0$; (B) the constrained region of ϕ-space (red solid tetrahedron) and its mapping back to θ-space (black wire tetrahedron) for the case of an amplicon with $n = 2$ CpG site and read-error rate $\eta = 0$; and (C) the same as (A) with $\eta > 0$.

The situation when $\eta > 0$ is more complicated (see Figure 1C for the case of $n = 1$ CpG site). In principle, an observed distribution y_i/n_{read} can be skewed either towards the fully unmethylated pattern $(0, \ldots, 0)$ or the fully methylated pattern $(1, \ldots, 1)$ by the transformation M^{-1}. In practice, however, and particularly in the case of non-vertebrates, observed distributions are typically weighted in favour of the fully unmethylated pattern and patterns within a small Hamming distance of the fully unmethylated pattern. Such distributions will be skewed towards the fully unmethylated pattern and the number of reported patterns will decrease, as observed in Figure 3 of the main paper.

References

[1] Akman, K., Haaf, T., Gravina, S., Vijg, J. and Tresch, A. (2014) Genome-wide quantitative analysis of DNA methylation from bisulphite sequencing data, *Bioinformatics*, Advance access publication. doi:10.1093/bioinformatics/btu142
